# Supplementary material for: Innovative Pre-Clinical Data Using Peptides to Intervene in the Evolution of Pulmonary Fibrosis
Source: Int J Mol Sci. 2023 Jul 4;24(13):11049. doi: 10.3390/ijms241311049 (PMC10341510; doi:10.3390/ijms241311049)
Supplement: Supplementary file 1 [file ijms-24-11049-s001.zip › ijms-2434027-supplementary.pdf]

Supplementary Figures

Supplementary Figure S1: Histological analysis of pulmonary tissue of BLM-instillated BALB/c mice

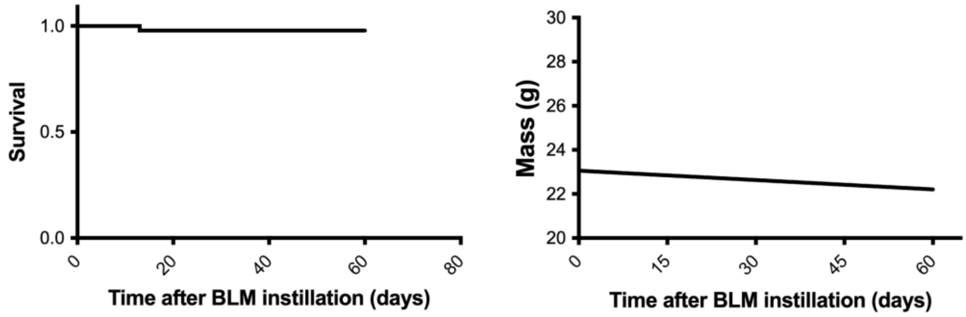

Figure S1. Survival and body mass of BLM-instilled mice over time.

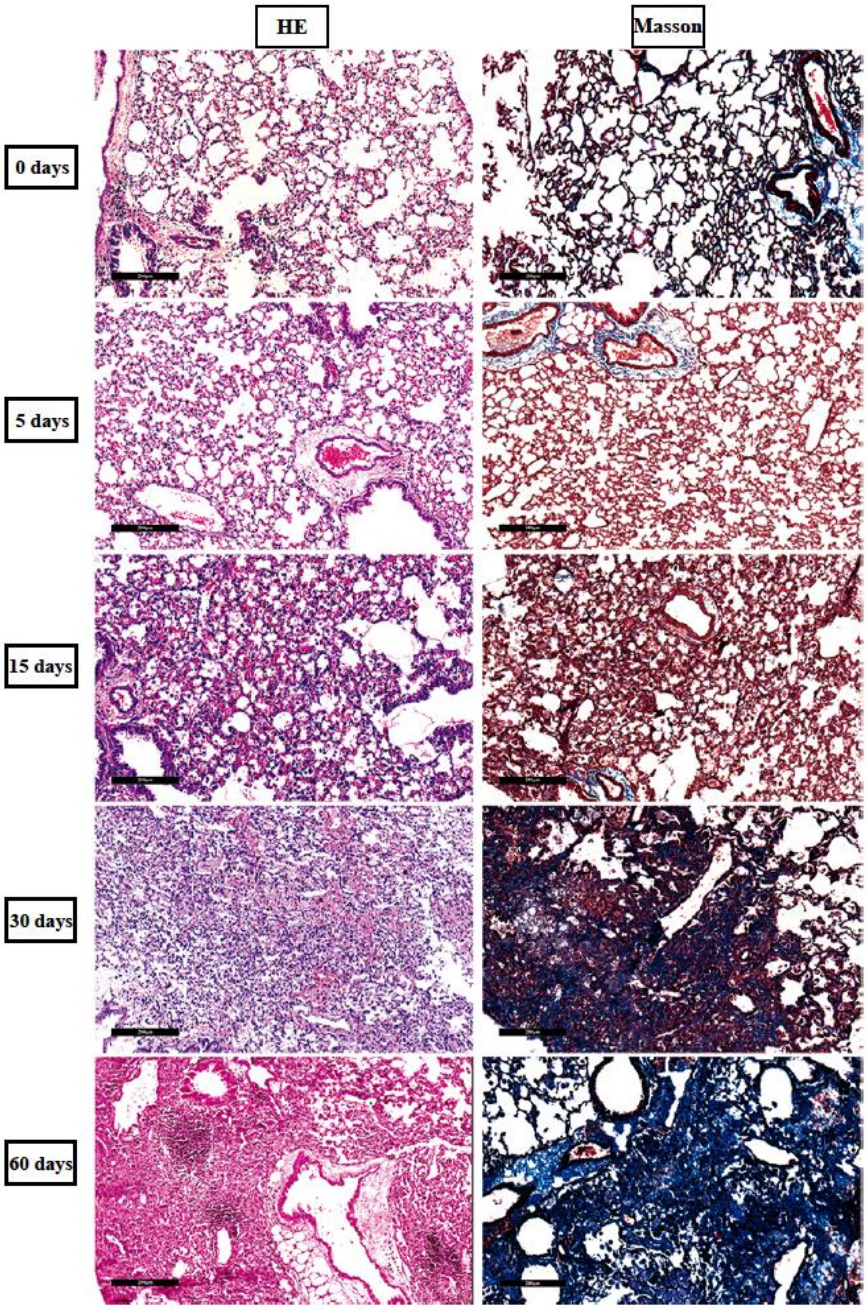

.

Figure S2. Histological analysis of pulmonary tissue of BLM-instilled BALB/c mice.

A

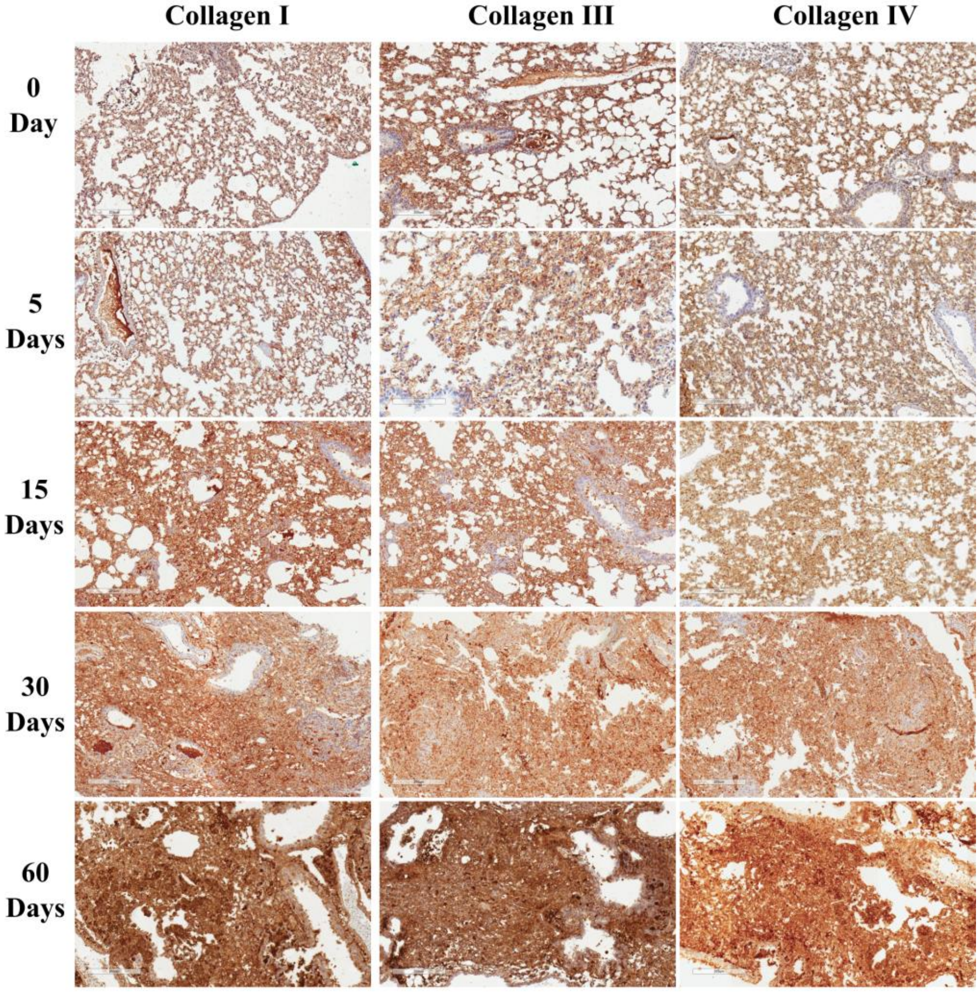

B

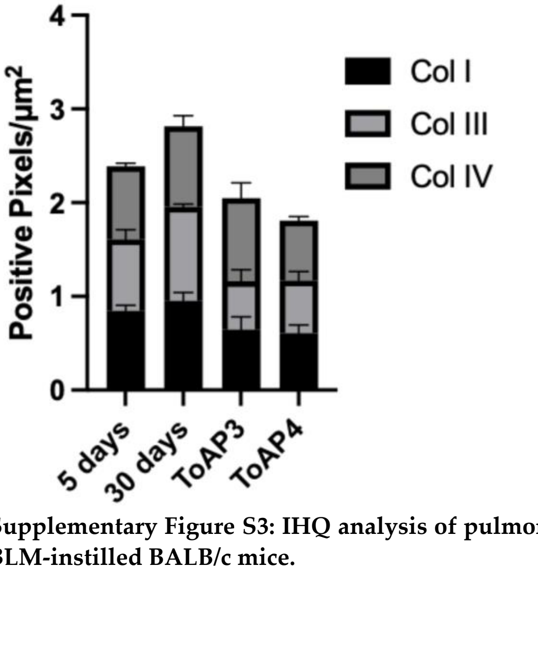

Supplementary Figure S3: IHQ analysis of pulmonary tissue collected from BLM-instilled BALB/c mice.

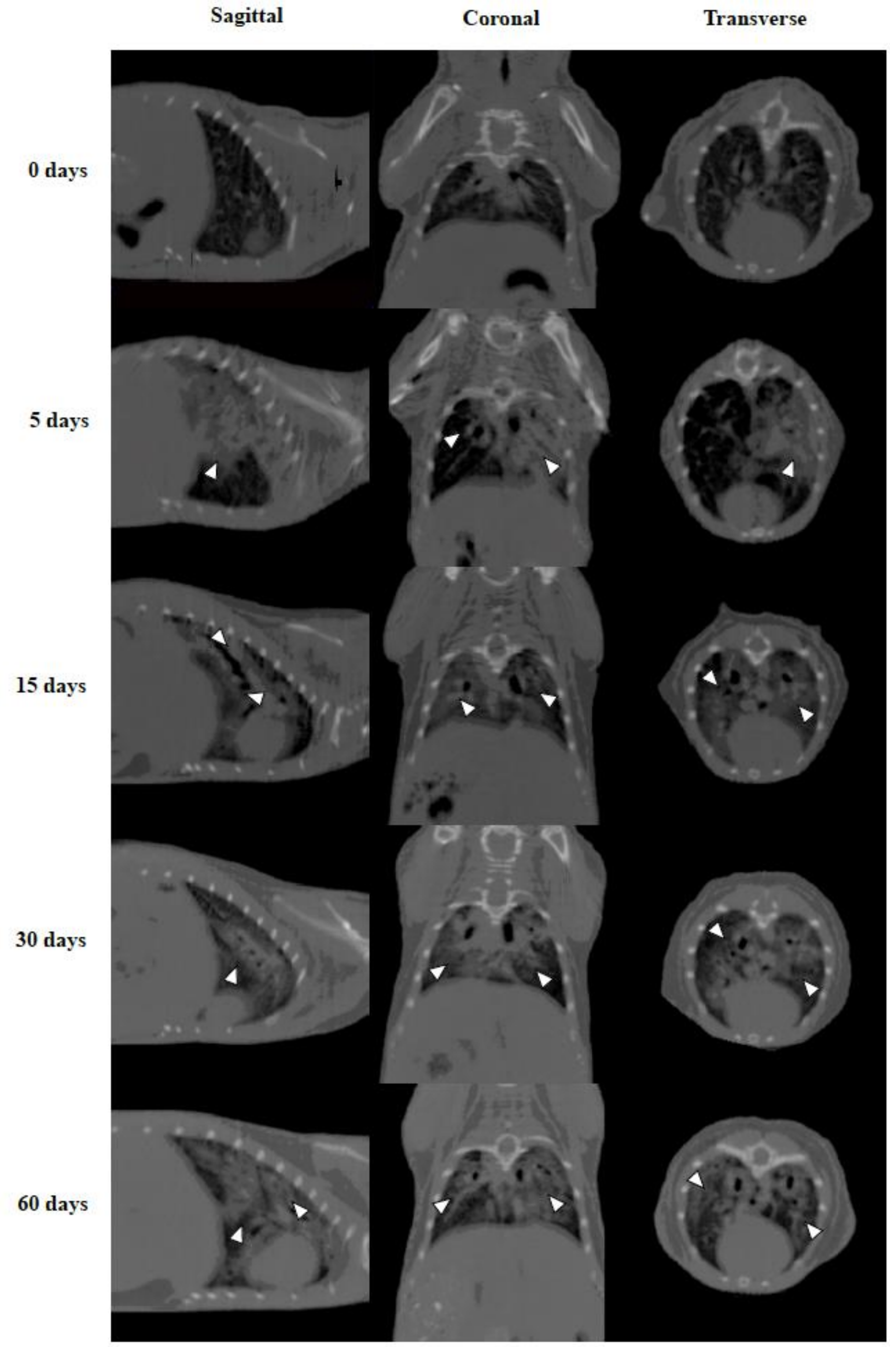

Supplementary Figure S4: μCT images of BLM-instilled BALB/c mice. White arrowheads show areas of opacification that suggest fibrosis formation.

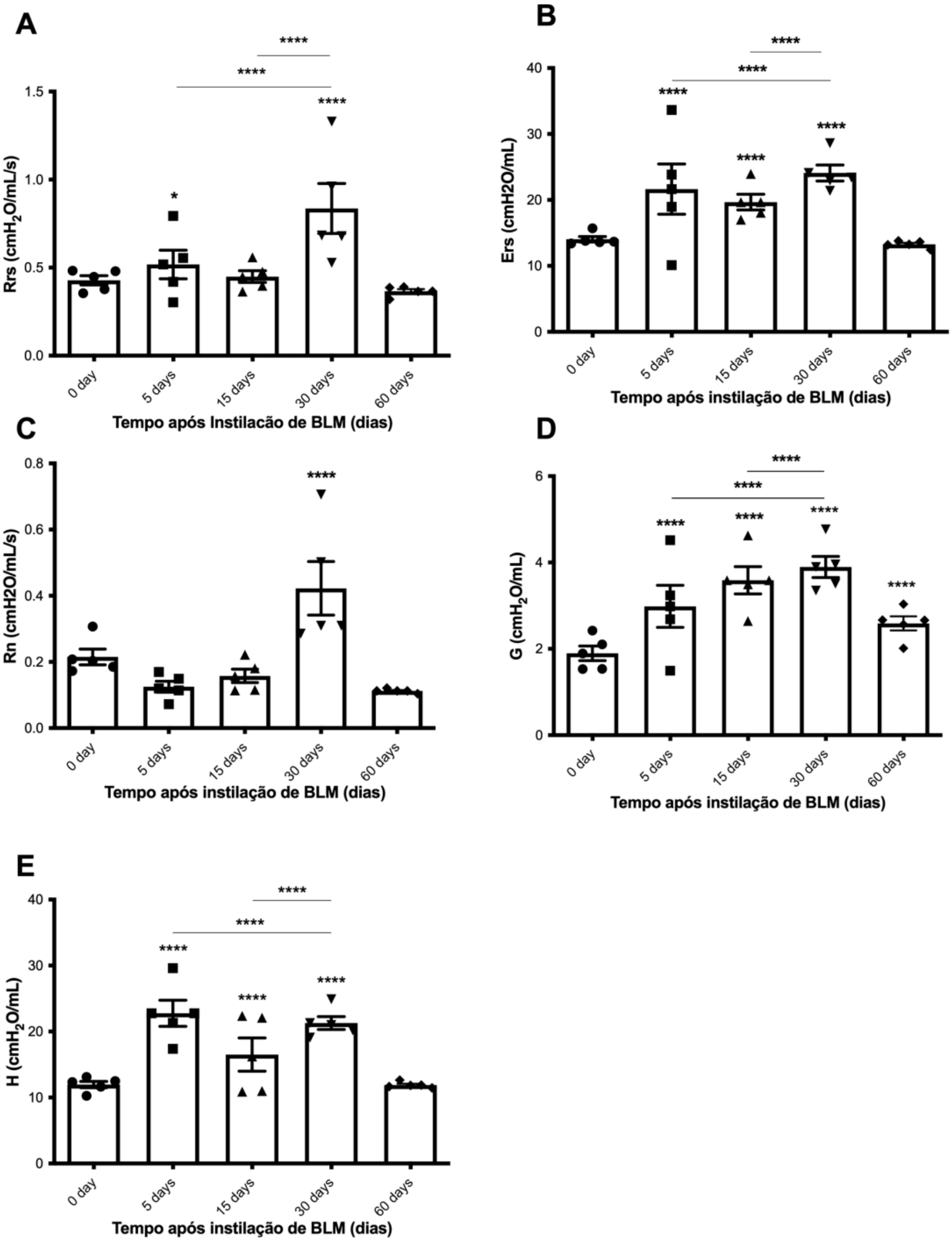

Supplementary Figure S5: Respiratory mechanics of BLM-instilled BALB/c mice. *P < 0.05, ****P < 0.0001.
